# The Impact of Persevering Home Full-Body In-Bed Gym Exercise on Body Muscles in Aging: A Case Report by Quantitative Radio-Densitometric Study Using 3D and 2D Color CT

**DOI:** 10.3390/diagnostics14242808

**Published:** 2024-12-13

**Authors:** Riccardo Forni, Paolo Gargiulo, Gabriele Boretti, Marco Quadrelli, Tommaso Baccaglini, Aldo Morra, Barbara Ravara, Sandra Zampieri, Amber Pond, Ugo Carraro, Maria Chiara Maccarone, Stefano Masiero

**Affiliations:** 1 Institute of Biomedical and Neural Engineering, Reykjavik University, 101 Reykjavik, Iceland; riccardo21@ru.is (R.F.); paolo@ru.is (P.G.); gabrieleb@ru.is (G.B.); 2 Landspitali, University Hospital of Iceland, 101 Reykjavik, Iceland; 3 Synlab Euganea Medica, 35020 Padua, Italy; dr.marcoquadrelli@gmail.com (M.Q.); tommaso.baccaglini@synlab.it (T.B.); aldo.morra@synlab.it (A.M.); 4 Synlab IRCCS SDN S.p.A., 80143 Naples, Italy; 5 Department of Biomedical Sciences, University of Padova, 35131 Padua, Italy; barbara.ravara@unipd.it (B.R.); sanzamp@unipd.it (S.Z.); ugo.carraro@unipd.it (U.C.); 6 Department of Surgery, Oncology and Gastroenterology, University of Padova, 35131 Padua, Italy; 7 CIR-Myo-Interdepartmental Research Center of Myology, University of Padova, 35131 Padua, Italy; stef.masiero@unipd.it; 8 A&C M-C Foundation for Translational Myology, 35100 Padua, Italy; 9 Anatomy Department, Southern Illinois University, Carbondale, IL 62901, USA; apond@siumed.edu; 10 Physical Medicine and Rehabilitation School, University of Padova, 35128 Padua, Italy; 11 Department of Neuroscience, Section of Rehabilitation, University of Padova, 35128 Padua, Italy

**Keywords:** aging, computed tomography, home Full-Body In-Bed exercise, muscle quality, radiodensitometry, sarcopenia

## Abstract

Background and Clinical Significance: Sarcopenia, characterized by muscle loss and fat infiltration, poses a significant health burden for aging populations. Quantitative Color 2D and 3D radiodensitometry provides a powerful tool to monitor muscle quality and quantity through CT imaging. This study assessed the impact of a ten-year-long home-bed gym exercise intervention on muscle quality in an elderly subject using CT-derived radiodensitometric analysis. The study involved two comparative analyses: Study A, which compared knee-to-ankle CT scans of the subject between 2013 and 2023; and Study B, which compared the subject’s 2023 thigh CT scan with a cohort of 2500 elderly Icelandic individuals from the AGES-Reykjavik study. Case Presentation: A 70-year-old male began a home-based Full-Body In-Bed Gym exercise program in 2013. Quantitative muscle volume and radiodensity measurements were performed using CT at baseline and after ten years. Results: Study A shows significant improvements in muscle volume observed in the knee-to-ankle region, while a slower decline in radiodensity was noted, indicating substantial preservation of muscle quality despite the expected decay of ten-year aging. For instance, muscle volume increased by 15% in the left Soleus muscle and by 6% in the right Soleus muscle, while the average radiodensity decreased by 12–17 HU. The subject’s thigh muscle quality at 80-years-old is above the AGES-Reykjavik’s cohort average, with reduced fat infiltration. Conclusions: Long-term home Full-Body In-Bed Gym, a low-impact exercise, can mitigate aging sarcopenia, as evidenced by improved tissue radiodensity and muscle mass substantial preservation. This suggests potential applications in personalized healthcare strategies to enhance muscle preservation among aging populations.

## 1. Introduction

Sarcopenia, the age-related decline in muscle strength and mass, significantly impacts the elderly population’s quality of life, increasing risks of falls, frailty, and mortality [1].

Studies demonstrate that maintaining moderate to high levels of physical activity throughout life is the most effective countermeasure against age-related decline in muscle health and function [1]. However, a significant portion of the global population remains largely sedentary, a trend that only intensifies with age. This widespread lack of physical activity accelerates the natural decline associated with aging, contributing to conditions like sarcopenia, reduced mobility, and overall poorer quality of life in older adults [2].

As a result of the growing interest in diagnosing and monitoring sarcopenia, quantitative radiodensitometry using computed tomography (CT), which measures tissue density based on its attenuation of X-rays during a CT scan, has become a valuable and reliable tool. This technique allows for a detailed assessment of muscle quality, including muscle density and the degree of fat infiltration, providing critical insights into both muscle mass and structural integrity [3].

This study addresses a significant research gap by providing long-term evidence about the effectiveness of accessible home-based exercise programs, specifically the Full-Body In-Bed Gym, in mitigating sarcopenia in elderly populations. The Full-Body In-Bed Gym is a home-based exercise protocol designed to address muscle weakness and atrophy, particularly in aging or impaired populations [4,5,6]. Inspired by established cardiovascular and respiratory rehabilitation protocols for surgical patients, this regimen adapts those principles into a short (10–20 min) sequence of 10–15 simple, tool-free exercises that can be performed entirely in bed. The exercises include movements such as hand gripping, ankle flexion, arm and leg cycling, spinal stretching, and progressive weight-bearing activities, all aimed at enhancing muscle strength, mobility, and overall physical fitness. Starting with as few as 3–5 repetitions, participants gradually increase to 15–20 repetitions as they build endurance. Over time, the speed and intensity of exercises are adjusted to maximize their benefits.

The program has been shown to be effective in improving quality of life and reducing pain in elderly individuals, with participants reporting significant enhancements after two months of consistent practice. Furthermore, this program not only offers a practical method for preserving muscle mass and function in the elderly, but it also provides a significant exercise opportunity for economically disadvantaged populations, who may not be able to afford expensive equipment or gym memberships [4,5,6].

Despite the growing recognition of exercise as a key intervention to combat muscle loss, there is limited data on the sustained impact of such programs. Here, we explore the application of quantitative radiodensitometry paired with 2D and 3D imaging to acquire information about the average X-ray absorption properties of soft tissue and their distributions as Hounsfield Units (HU). Indeed, while quantitative radiodensitometry, including 2D and 3D imaging, has shown promise in assessing muscle health, its application in aging populations has not been well explored.

This case report aimed to fill these gaps by utilizing a 10-year longitudinal study with CT imaging to monitor muscle health outcomes, offering a cost-effective and accessible solution for preserving muscle mass and improving quality of life in older adults.

The aim of this case report was twofold: (1) to support the fact that the Full-Body In-Bed Gym program can combat sarcopenia; and (2) to provide further evidence supporting the effectiveness of 2D and 3D quantitative radiodensitometry as a monitoring tool for muscle health in aging populations.

## 2. Case Presentation

### 2.1. Study Design

This study focused on a 70-year-old male subject who undertook a home-based exercise regimen for over 10 years. The main objectives were to assess changes in muscle volume and quality over time using radiodensitometry. The study was divided into two parts: (1) Study A compared CT scans of the knee-to-ankle region from 2013 and then 2023; while (2) Study B compared data from a cross-section of a 2023 thigh CT scan with the data from a cohort of 2700 age-matched individuals from the AGES-Reykjavik study.

### 2.2. Intervention

The home-based Full-Body In-Bed Gym program employed here consisted of 10 exercises performed six times per week. The Full-Body In-Bed Gym program allows individuals to engage in various exercises from the comfort of their beds, making it particularly appealing for those with mobility limitations or those who prefer to exercise without getting out of bed. This program typically involves a series of low-impact exercises that can be performed while lying down, sitting, or even standing next to the bed. Exercises may include strength training and stretching activities. The subject performed 10 repetitions of each exercise starting in 2013, which he gradually increased in intensity and volume over time. Each session lasted between 5 and 15 min.

**Figure 1:**
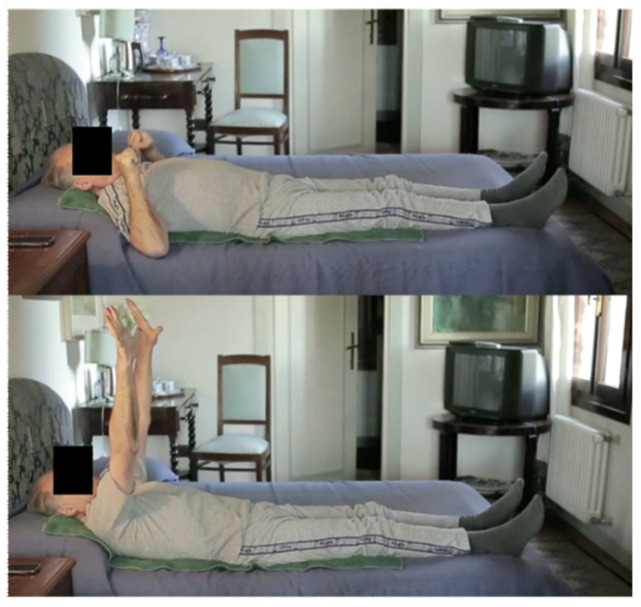
An example of the ten exercises which constitute the home-based Full-Body In-Bed Gym protocol.

Figure 1 shows one of the ten home Full-Body In-Bed Gym protocol exercises. The figure was reprinted with permission from the reference Maccarone et al. 2023 [4].

### 2.3. Imaging Protocol

#### 2.3.1. Study A: Knee-to-Ankle Comparison (2013 vs. 2023)

CT scans were conducted using a Philips iCT 256 scanner. Baseline scans from 2013 were compared to scans from 2023, focusing on the region from the knee to the ankle. Both scans were acquired at 120 kVp, with a voxel size of 0.665 mm and slice thickness of 0.335 mm. Radiodensity and muscle volume were measured using custom segmentation software, analyzing three major muscle groups: *Tibialis anterior*, *Soleus*, and *Gastrocnemius* muscles.

#### 2.3.2. Study B: Thigh Comparison: Longitudinal Case Study (2013–2023) vs. AGES-Reykjavik Population [7,8]

Images were captured from the mid-thigh region using a voltage of 120 kVp. This study compared the 2023 CT scan of the subject’s thigh to those of a reference population of 2500 age-matched individuals from the AGES-Reykjavik cohort. Radiodensity was measured for fat (−200 to −10 HU), connective (−10 to 40 HU), and muscle (40 to 200 HU) tissues. The case study values were plotted against the AGES-Reykjavik cohort group to assess relative muscle quality and fat infiltration.

### 2.4. Medical History of the Octogenarian

The case study subject was a male, born in Abano Terme, Padua, Italy, on 23 February 1943. His medical history was collected at the beginning of the study, with the main pathological events including:

In 1969, the subject experienced a vehicular accident that resulted in fractures to both legs and ankles. A significant complication from the incident was the rupture of his spleen, which necessitated surgical removal to address the internal bleeding. He survived as a result of the intravenous infusion of fluids and blood from donors. Unfortunately, during his subsequent convalescence, he developed transfusion-induced hepatitis, which delayed the recovery of the skeletal lesions.

Over the next 40 years, he experienced only two episodes of viral flu and has not developed any COVID-19 infections to date. He has received five doses of anti-COVID vaccines.

Despite having no family history of hypertension and only passive smoking exposure, he was diagnosed with asymptomatic arterial hypertension at the age of 40. He delayed treatment, which ultimately resulted in severe coronary artery disease.

From his medical history recently collected at the Hospital of Rovigo, Italy, we report the following:

An 80-year-old man with a history of hypertension and mixed dyslipidemia, treated pharmacologically since age 45, began experiencing symptomatic effort angina at age 68. Coronary angiography revealed significant disease in the right coronary artery (RCA), left circumflex artery (LCx), and first diagonal branch (D1), leading to percutaneous coronary intervention (PCI) and a drug-eluting stent (DES) placement. Three years later, he developed a symptomatic popliteal artery aneurysm requiring surgical exclusion. At a 10-year follow-up, a transthoracic echocardiogram indicated chronic ischemic/hypertensive cardiomyopathy with preserved left ventricular function. Holter monitoring showed initial conduction system impairment, including first- and second-degree atrioventricular blocks and frequent ectopic beats. Follow-up coronary angiography demonstrated good patency of the previously implanted DES. His optimized outpatient cardiology regimen included aspirin 100 mg, Irbesartan 300 mg once a day, calcium antagonist 10 mg once a day, and rosuvastatin 10 mg.

Despite this strong evidence of a slowly progressive cardiomyopathy, the subject still performs all regular activities of daily living, engages in light voluntary exercise (home-based Full-Body In-Bed Gym), and undertakes heavy gardening work.

### 2.5. Quantitative Image Analysis

**Figure 2:**
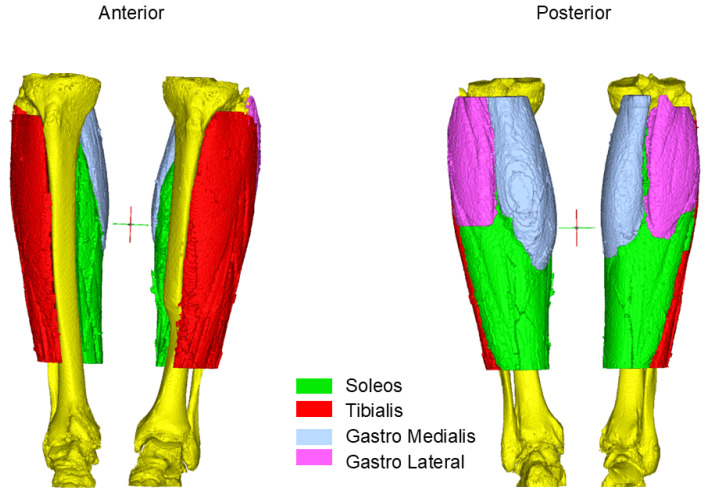
Muscle segmentation of the Octogenarian: Different region of interest from the first time point in 2013 when he was 70-years-old.

In Study A we compared volumetric CT data taken from knee-to-ankle joints (Figure 2).

CT images were analyzed using Mimics 26.0 segmentation software (Materialize, Belgium).

To assess the effect of the intervention, a 3D model of the calves was reconstructed from the two datasets. An initial thresholding between 0 and 150 HU was performed. To regularize the borders and eliminate floating pixels, a closing gap operation with a gap size of 4 pixels followed by an erosion of 2 pixels was performed. These operations resulted in a 3D model of the muscles in the calves, including the intramuscular fat and other tissues inside muscle bundles. The four principal muscles were separated with a manual splitting tool following their borders over the darker background, and the volume of each bundle was extracted. At this point, the histogram of each muscle was drawn, and the HU spanned from −200 to 200. The contribution of different tissue types was extracted: fat (−200 to −10 HU), connective tissue (−10 to 40 HU), and muscle (40 to 200 HU) [9]. The average HU was calculated and stored for further comparison.

**Figure 3:**
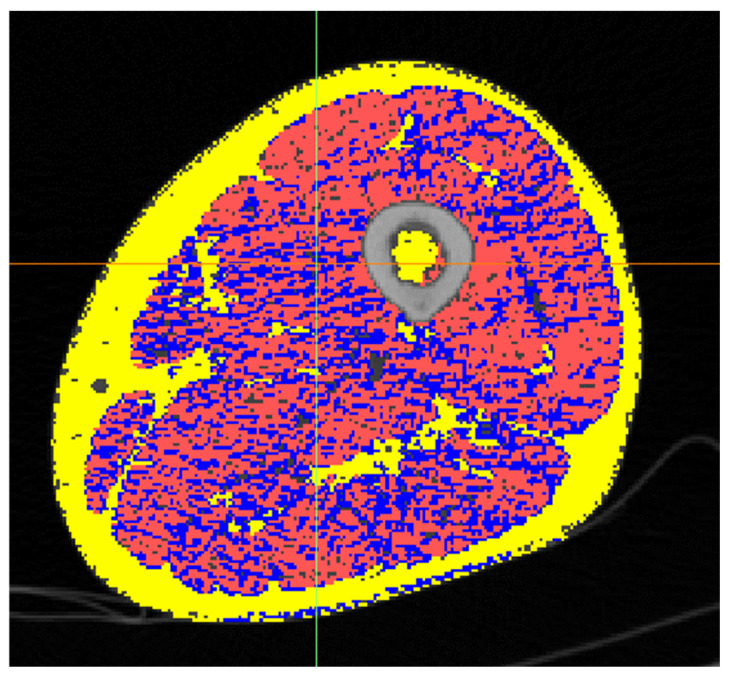
Cross-section of the mid-thigh of the Octogenarian. Fat in yellow, connective in blue and muscle in red.

**Figure 4:**
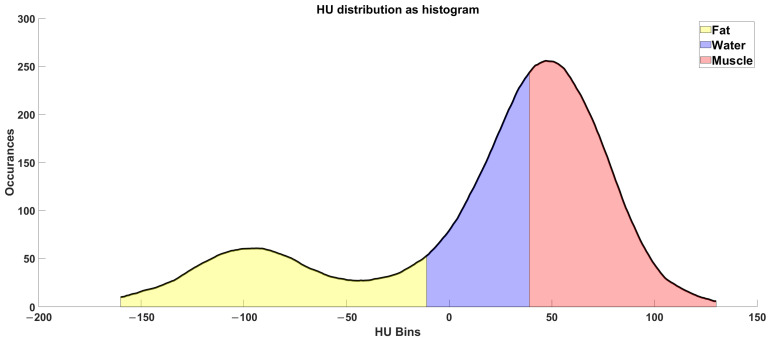
Radio densiometric profile of the cross-section of the mid-thigh of the Octogenarian in 2023, where the areas of fat, connective, and muscle tissues are colored, respectively, in yellow, blue and red.

In Study B, we analyzed a mid-thigh CT image cross-section of the Octogenarian (Figure 3). We calculated the average radiodensity of connective, muscle, and fat tissues, extracted with the same threshold introduced before (Figure 4), and compared it with age-matched controls from the AGES-Reykjavik cohort database. The amount of fat computed in this manner evaluated both intramuscular and subcutaneous fat. In the AGES cohort, we analyzed the same parameters from 2700 individuals aged 65 to 95 [7,8].

### 2.6. Results

#### 2.6.1. Study A: Knee-to-Ankle Muscle Volume and Density Changes (2013 vs. 2023)

Data analysis revealed that muscle volume increased across all studied groups of muscles within the distal part of the leg, particularly in the *Soleus* and *Tibialis anterior* muscles. The left *Soleus* muscle showed a 15% increase, while the right *Soleus* muscle increased by 6% (Table 1). Despite the increases in muscle volume, the muscle radiodensity decreased (Table 2); however, this decline occurred at a slower pace than reported in the literature about the average muscle radiodensity decay that accompanies 10 years of aging (see discussion). Indeed, the radiodensity data revealed a moderate decline in muscle density and increased fat infiltration. Nonetheless, the fat infiltration of the subject remained lower than that reported in the literature [10,11,12,13,14,15,16,17,18,19,20].

**Figure 5:**
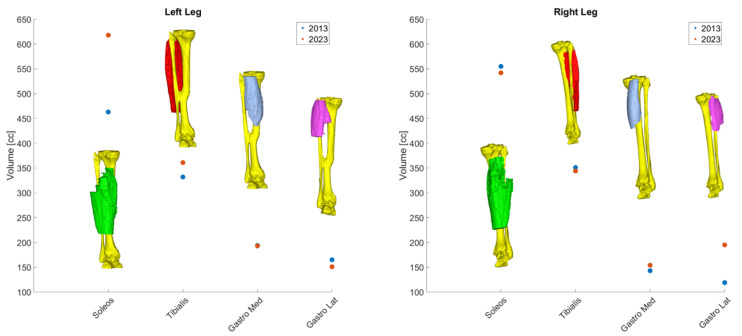
Volumetric changes in muscular bundle by leg.

The analysis also revealed that total muscle volume increased in both legs over the ten years of intervention (15% left leg and 6% right leg). However, not all muscles underwent the same changes: left leg distal muscles (*Soleus* and *Tibialis* muscles) experienced an increase in volume, whereas on the right leg their volume slightly decreased (Table 1, rows 1 to 4). On the other hand, an opposite scenario was revealed for the *Gastrocnemius* muscles. The left volume decreased less than 10% in volume while the right *Gastrocnemius* muscle gained almost 65% in volume laterally (Table 1, rows 5 to 8). A graphical representation of these results is pictured in Figure 5.

On the other hand, average muscle radiodensity increased from 12 to 17 HU, whereas the average fat and connective tissues radiodensity increased from 5 to 17 HU.

#### 2.6.2. Study B: Thigh Muscle Quality Compared to AGES-Reykjavik Population

**Figure 6:**
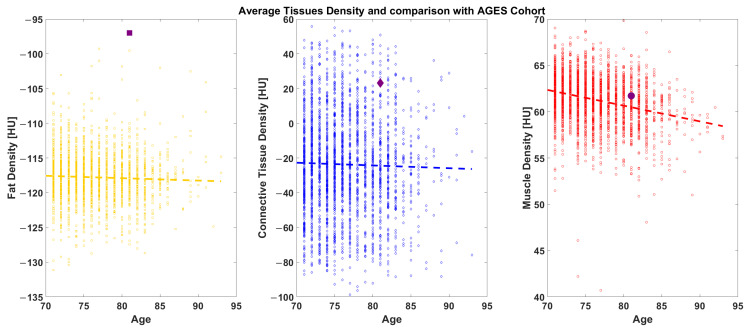
AGES comparison: The Octogenarian (reported as a black symbol) is shown against a cohort of 2700 age-matched individuals ranging from 70 to 95-years-old.

The radiodensitometric profile extracted from the cross-section of the midthigh is reported in Figure 6. The different tissues are highlighted with different colors. The extracted averages are 61.25 HU for muscle (red), 23.20 HU for connective tissue (blue), and −81.70 HU for fat (yellow).

Compared to the AGES-Reykjavik population, the Octogenarian’s 2023 thigh CT scan revealed a higher-than-average muscle density and reduced fat infiltration. Indeed, when the subject’s average values are plotted with that of the AGES population, the data place the subject above average in muscular density and at a lower subcutaneous fat density (Figure 6).

## 3. Discussion

This study demonstrated the effectiveness of a long-term, home-based exercise protocol in mitigating sarcopenia, as evidenced by the preservation of muscle volume and the reduced fat infiltration in the muscle of our exercising elderly subject. Quantitative radiodensitometry using CT imaging has proven to be a valuable tool for assessing long-term muscle quality and quantity.

The home-based Full-Body In-Bed Gym program demonstrated significant benefits in preserving muscle mass and quality. The subject was in his seventies (“Septuagenarian”) when the intervention began and is now in his eighties (“Octogenarian”). The unbalanced volume changes (i.e., differences in left and right legs), reported in Figure 4, may be a consequence of the right-side dominancy of the subject’s posture.

Additionally, the Full-Body In-Bed Gym program contributed to improved quality of life, reduced pain, and a decreased risk of sarcopenia in the elderly sedentary individual [4]. This suggests that similar exercise programs could be effective for a broader aging population. An added benefit of the home-based Full-Body In-Bed Gym is that this safe approach is highly cost-effective.

Compared to data in the literature, the subject maintained higher than average radiodensity values and better muscle quality, highlighting the potential of persevering low-medium intensity exercise to slow muscle decay associated with aging. Our findings are consistent with previous research, showing the efficacy of exercise interventions in combatting sarcopenia, whether due to aging and neuromuscular disorders [10,11,12,13,14,15,16,17,18,19,20], or tumors [21,22,23]. Specifically, quantitative radiodensitometry has proven to be a reliable method for assessing both muscle mass and quality, allowing for precise monitoring of age-related and exercise-related changes.

Muscle type also plays an important role in age-related muscle degeneration. Age-related atrophy has proven to affect various muscles fibers differently. For example, type II fibers, which are more prevalent in the locomotor muscles of the lower body (e.g., *Gastrocnemius* muscle), experience greater rates of atrophic change while only moderate changes occur among type I fibers, characteristic of the muscles involved in a postural role (e.g., *Soleus* muscle) [24,25,26,27]. On the other hand, hypoplasia has also been found to contribute partially to muscle mass reduction [24]. Some reports suggest that hypoplasia and atrophy contribute equally to muscle loss [27]. Indeed, studies have highlighted the critical importance of both processes, although there remains some controversy over which one plays the more significant role [28,29,30,31,32,33,34,35,36,37,38,39].

Similar results were found in a previous study: Ikezoe et al. (2011) found that muscle thickness, measured by ultrasound, decreases with aging based on the type of muscle, and that life-space assessment (LSA) correlates only with the *Soleus* muscle [40]. Reimers et al. (2012) demonstrated the impact of aging and athletic activity on a large, mixed cohort. They found that in athletes, calf thickness remained stable with age, although fat content increased. However, in non-athletic males, the thickness of the *Gastrocnemius* and *Soleus* muscles decreased significantly—by 16% between the ages of 20 and 70—while the *Tibialis* muscle showed only a 7% reduction [41]. On the other hand, Trappe et al. (1996) reported that elite distance runners and lesser trained/untrained subjects have the same calf cross-sectional area [42]. In addition, Klitgaard et al. (1990) found that elderly swimmers and runners had similar calf cross-sectional areas compared to an age-matched less active control group [43].

Exercise plays a crucial role in the prevention and management of sarcopenia. Numerous studies in the literature demonstrate that regular exercise programs, particularly resistance and strength training, can significantly reduce the risk of sarcopenia by improving muscle mass, strength, and mobility [44]. However, in low-income countries, where access to fitness facilities and equipment is limited, the challenge lies in implementing sustainable and accessible interventions [45,46,47,48,49,50]. Low-cost solutions, such as bodyweight exercises or home-based programs, have proven effective in promoting muscle health even in resource-limited settings [7,51,52,53]. Simple, safe, and adaptable protocols, such as the Full-Body In-Bed Gym, could serve as valuable strategies to combat sarcopenia and enhance quality of life, particularly in economically vulnerable populations.

Future research should expand on these results by studying more extensive cohorts and incorporating diverse exercise regimens to validate the benefits of such interventions further [2]. Additionally, the role of quantitative imaging in tracking muscle health warrants further exploration to enhance diagnostic and therapeutic strategies for sarcopenia.

## 4. Conclusions

CT-based quantitative radiodensitometry is a valuable method for tracking muscle health over time, particularly in aging populations. The Full-Body In-Bed Gym program, designed for home use, showed promising results in maintaining muscle mass and quality, which could contribute to improved quality of life and reduced sarcopenia risk among sedentary older adults. These findings highlight the potential for similar low-cost exercise programs to serve as practical and scalable interventions, benefiting both individual health outcomes and societal healthcare systems. Furthermore, quantitative radiodensitometry paired with 2D and 3D imaging is an excellent way to assess muscle quality and would be useful for diagnoses of muscle pathologies and follow-ups of muscle treatment regimes.

## Figures and Tables

**Table 1:** Muscles bundle volumes and their percent change in response to the intervention.

Row	Muscle Group	Vol 70 ys (cm^3^)	Vol at 80 ys (cm^3^)	% Change
1	*Soleus* (Left)	463	618	+33%
2	*Soleus* (Right)	555	542	−3%
3	*Tibialis* (Left)	332	361	+9%
4	*Tibialis* (Right)	351	344	−2%
5	*Grastoc* Medial (Left)	194	194	/
6	*Grastoc* Medial (Right)	143	154	+8%
7	*Grastco* Lateral (Left)	165	151	−8%
8	*Grastoc* Lateral (Right)	119	195	+64%

**Table 2:** Tissue radiodensity by muscular bundle before (rows 1–4) and after (rows 5–8) the intervention. Values in HU.

Row	Left Leg (70 Years)	Muscles	Fat	Connective	Right Leg (70 Years)	Muscles	Fat	Connective
1	*Tibialis*	68	−35	23	*Tibialis*	71	−33	22
2	*Soleus*	71	−38	21	*Soleus*	69	−37	21
3	*Gastrocnemius medial*	73	−38	21	*Gastroc*_*medial*	70	−37	23
4	*Gastrocnemius lateral*	69	−38	21	*Gastroc*_*lateral*	68	−32	21
**Row**	**Left Leg (80 Years)**	**Muscles**	**Fat**	**Connective**	**Right Leg (80 Years)**	**Muscles**	**Fat**	**Connective**
5	*Tibialis*	56	−24	27	*Tibialis*	57	−21	29
6	*Soleus*	58	−28	23	*Soleus*	57	−26	26
7	*Gastrocnemius medial*	56	−27	25	*Gastroc_medial*	55	−28	26
8	*Gastrocnemius lateral*	55	−25	25	*Gastroc_lateral*	54	−25	25

## Data Availability

Detailed data supporting the findings of this study can be obtained from the corresponding author.
